# Enhancing Protein A performance in mAb processing: A method to reduce and rapidly evaluate host cell DNA levels during primary clarification

**DOI:** 10.1002/btpr.2882

**Published:** 2019-08-13

**Authors:** Kenneth C. Koehler, Zona Jokondo, Janani Narayan, Alexei M. Voloshin, Angelines A. Castro‐Forero

**Affiliations:** ^1^ 3M Separation and Purification Sciences Saint Paul Minnesota; ^2^ Johns Hopkins University, Chemical and Biological Engineering Baltimore Maryland

**Keywords:** depth filtration, DNA reduction, Emphaze AEX Hybrid Purifier, Protein A chromatography, turbidity

## Abstract

The use and impact of 3M™ Emphaze™ AEX Hybrid Purifier, a single‐use, fully synthetic chromatographic product, was explored to reduce host cell DNA (HC‐DNA) concentration during the primary clarification of a monoclonal antibody (mAb). An approximately 5‐log reduction in HC‐DNA was achieved at an Emphaze AEX Hybrid Purifier throughput of 200 L/m^2^. The appreciable reduction in HC‐DNA achieved during primary clarification enhanced Protein A chromatography performance, resulting in a sharper and narrower elution profile. In addition, a 24× improvement in host cell protein (HCP) removal and fewer impurities nonspecifically bound to the Protein A column were observed compared to those resulting from the use of depth filtration for clarification. The use of a rapid, qualitative acidification assay to facilitate HC‐DNA monitoring was also investigated. This assay involves the acidification‐induced precipitation of HC‐DNA, enabling the easy and rapid detection of DNA breakthrough across purification media such as Emphaze AEX Hybrid Purifier by means of turbidimetric and particle size measurements.

## 1 INTRODUCTION

The modern production of monoclonal antibodies is a complex, multistep process requiring the interplay between many different unit operations to purify the target product with good yield and high purity.^1^, ^2^, ^3^, ^4^, ^5^, ^6^ Traditionally, monoclonal antibody (mAb) production has been divided into two distinct stages, upstream processing and downstream purification, separated by a product capture step, typically Protein A chromatography.^3^, ^4^ Upstream processing generally entails cell line development, cell culture cultivation, and the clarification of harvested cell culture fluid (HCCF). During HCCF clarification, large insoluble debris, such as host cells, is removed. Depending upon HCCF characteristics, a single clarification step may suffice, or multiple unit operations in series, such as centrifugation followed by depth filtration, may be required to achieve the desired level of clarity. The clarified cell culture fluid (CCCF) produced during upstream processing is typically passed through a sterilizing grade membrane to remove any residual smaller debris and provide bioburden control before capture chromatography and downstream processing.

Protein A affinity chromatography commonly serves as the capture step in mAb production. The Fc region of a mAb exhibits a strong affinity and high selectivity toward Protein A, binding the target protein to the chromatography ligand or resin.^4^, ^7^, ^8^ During loading, the mAb is bound to the chromatography resin, whereas other impurities such as host cell proteins (HCPs) generally interact to a lesser extent with Protein A ligands and flow through the column to be discarded. After loading onto the Protein A column, the bound mAb is washed to remove residual HCPs and other contaminants. After washing, a change in the mobile phase pH results in elution of the target mAb molecule. Processing of the Protein A eluate then continues in the downstream unit operations to remove residual contaminants and further increase the target mAb purity.

This sequential mAb purification scheme establishes a cascade where the removal of impurities at one stage of the process directly impacts the performance of subsequent unit operations. Therefore, achieving a significant reduction in contaminants early in the process enables simpler, more efficient mAb production. Upstream clarification unit operations readily illustrate this concept by effectively removing macroscopic, insoluble debris such as host cells. However, soluble impurities such as chromatin and its HCP, DNA, and RNA constituents are not significantly reduced during primary clarification and are left for removal during downstream unit operations. As a result, downstream unit operations are often scaled to accommodate a high level and range of impurities, reducing their effectiveness. For instance, additional unit operations, such as the inclusion of ancillary depth filters and chromatography columns, must often be incorporated into downstream unit operations to achieve the target throughput and purity.^8^

One soluble contaminant in HCCF typically not cleared during primary clarification is chromatin, a complex of HC‐DNA coiled around histone proteins as well as RNA and other proteins.^9^, ^10^, ^11^, ^12^ The presence of chromatin has been documented to directly impact mAb purification and recovery, presenting a challenge to the affinity chromatography and downstream processing steps.^10^ In the case of Protein A chromatography, chromatin and HCPs have been found to nonspecifically bind to Protein A ligands, reducing the number of binding sites for the target mAb and effectively lowering the dynamic binding capacity of the affinity column.^10^, ^13^ In addition, chromatin has been shown to enable the coelution of HCPs with the target mAb from the Protein A column. Through interactions with chromatin, HCPs are not completely removed during the wash step and can “hitchhike” across the Protein A column and co‐elute with the desired mAb.^10^, ^11^

When HCPs and DNA are not completely removed during Protein A chromatography, the performance of downstream unit operations is also impacted. Failure to remove DNA can potentially lead to increased turbidity during neutralization after a low pH hold viral inactivation procedure, thus requiring the addition of a filtration step.^8^, ^14^

A number of chromatin‐directed clarification strategies have been proposed to address the presence of chromatin/DNA and the challenges it creates during and after Protein A chromatography. These approaches generally aim to significantly reduce chromatin levels during primary clarification or alter the operating conditions used during Protein A chromatography. Recent efforts have focused on selective precipitation to isolate and remove chromatin during primary clarification. In this case, chromatin precipitation is typically achieved through the addition of fatty acids, such as caproic or octanoic acid, allantoin or ethacrine, to CCCF.^9^, ^10^, ^11^, ^12^, ^15^

Rather than inducing the precipitation of soluble contaminants such as chromatin and DNA, other methods have incorporated the precipitation of the target mAb or target protein. A peptide or biopolymer featuring a Protein A epitope is introduced to the cell culture and binds the desired mAb, subsequently inducing precipitation. Upon isolation and washing of the precipitate, the mAb can be released into solution by pH modulation, allowing further purification.^16^, ^17^

Other methods to overcome the challenges chromatin/DNA presents to Protein A chromatography involve modifying the operating conditions for Protein A chromatography itself. One strategy used chromatofocusing using a pH gradient through the Protein A column to reduce HCP coelution with the target mAb.^18^ An alternative approach made use of two distinct flow rates, high and low, to influence residence time and minimize nonspecific binding and HCP hitchhiking as well as to improve overall column utilization.^19^ Changes to the column washing strategy have also been considered to improve HCP and other contaminant clearance across Protein A chromatography. Washes introducing additives that disrupt protein–protein interactions, such as urea, isopropanol, elevated pH, and increased salt concentration, have shown promise in reducing HCP levels in Protein A eluate.^14^, ^20^

Rather than modifying HCCF through the addition of extraneous material or altering the Protein A elution conditions, this study targeted the removal of HC‐DNA, a common component of chromatin, with a single‐use, advanced anion exchange media: Emphaze AEX Hybrid Purifier. Comprising solely synthetic materials, Emphaze AEX Hybrid Purifier provides Q‐functional chemistry on a polypropylene nonwoven scaffold followed by a 0.2 μm asymmetric polyamide membrane.^21^ The high positive charge of Emphaze AEX Hybrid Purifier enables the removal of negatively charged soluble impurities, including HC‐DNA. In this investigation, Emphaze AEX Hybrid Purifier was incorporated upstream of Protein A following depth filtration to remove HC‐DNA during primary clarification.

To assess the performance of techniques for the removal of soluble impurities such as HC‐DNA, HCP, and chromatin, the levels of these contaminants in the cell culture fluid must be monitored. Quantitative assays such as Picogreen, qPCR, and/or ELISA are typically conducted on aliquots collected over the course of purification to measure their concentration. These assays provide a means to accurately and quantitatively determine the effectiveness of HC‐DNA removal for a given solution; however, they are often costly and time consuming. Furthermore, multiple fractions may be collected and submitted for analysis but are typically not processed or analyzed until after the clarification process has been completed. With the challenge posed by current analytical techniques, the ability to rapidly identify the presence of significant soluble contaminant levels, such as HC‐DNA, during purification will aid in the development and design of primary clarification approaches that provide higher‐quality, standardized material to downstream unit operations. A rapid qualitative assay for HC‐DNA using turbidimetric readings and particle size analysis was explored during this investigation to facilitate the estimation of HC‐DNA reduction during primary clarification and potential breakthrough across purification media such as Emphaze AEX Hybrid Purifier.

## 2 EXPERIMENTAL

### 2.1 Cell culture conditions

A tociluzimab biosimilar feed stock was produced in Chinese Hamster Ovary (CHO) cells. The material was produced in a fed‐batch 50 L disposable stirred tank bioreactor (Eppendorf, Hamburg, Germany) up to a cell density of 7.7 × 10^6^ cells/mL with 74% final viability. The titer was approximately 4 g/L.

### 2.2 Cell culture harvest

**Figure 1:**
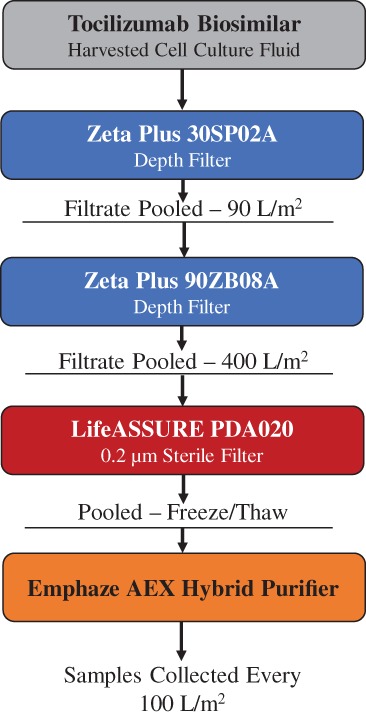
Overview of the tociluzimab harvest clarification process

Figure 1 provides a summary of the tociluzimab harvest clarification procedure. The bioreactor was harvested on day 14 using Zeta Plus™ 30SP02A as a primary filter at a throughput of 90 L/m^2^. The filtrate from 30SP02A was then processed using Zeta Plus 90ZB08A up to a throughout of 400 L/m^2^. After sterile filtration with LifeASSURE™ PDA020, this material was frozen at −80°C. The depth filtered material was thawed and then purified using Emphaze AEX Hybrid Purifier. Fractions were collected every 100 L/m^2^.

### 2.3 Protein A chromatography

An AKTA™ Avant chromatography system (GE Healthcare, Uppsala, Sweden) was used for all chromatography experiments. Protein A chromatography was performed using HiScreen MabSelect SuRe columns (GE Healthcare, Uppsala, Sweden) with a bed height of 10 cm and a column volume (CV) of 4.7 mL. The protocol used for all Protein A experiments was as follows: (a) equilibration, 5 CV of 20 mM phosphate pH 7.2, 150 mM NaCl at 300 cm/h; (b) loading, 30 mg/mL of tociluzimab at 200 cm/h; (c) wash: 5 CV of 20 mM phosphate pH 7.2, 150 mM NaCl at 300 cm/h; (d) elution: 3 CV of 50 mM sodium acetate, pH 3.5 at 200 cm/h; (e) acid stripping: 3 CV of 50 mM acetic acid pH 2.5 at 300 cm/h; (f) intermediate wash: 3 CV of 20 mM phosphate pH 7.2, 150 mM NaCl at 300 cm/h; (g) cleaning in place: 3 CV of 0.5 M NaOH, 15 min contact time; (h) re‐equilibration: 7 CV of 20 mM phosphate pH 7.2, 150 mM NaCl at 300 cm/h.

### 2.4 DNA, HCP, and mAb quantification

HC‐DNA was quantified by quantitative PCR (qPCR) using a resDNASEQ™ Quantitative CHO DNA Kit (Cat #440208, Thermo Fisher Scientific) according to the manufacturer's instructions. Host cell protein quantification was performed using a CHO HCP Kit, 3G (Cat #F550, Cygnus Technologies, NC) according to the manufacturer's instructions.

mAb quantification was performed by Protein A – HPLC. A POROS® A20 column, 0.1 mL (Thermo Fisher Scientific) was connected to an Agilent 1120 Series HPLC (Agilent, Santa Clara, CA). The sample injection volume was 50 μL. Equilibration was performed with 50 mM sodium phosphate pH 7.0 and 150 mM NaCl for 1 min. Elution was performed with 100 mM citrate, pH 2.5 for 1.5 min. The flow rate was 3 mL/min. The concentration was calculated using an in‐house IgG1 standard.

### 2.5 Turbidity and particle size analysis

Turbidity measurements were performed on sample aliquots of approximately 12 mL each using an ORION™ AQ4500 turbidity meter (Thermo Fisher Scientific). Dynamic light scattering (DLS) measurements were performed with a Microtrac Nanotrac Wave Flex (Microtrac) to characterize the particle size distributions in the samples collected.

## 3 RESULTS AND DISCUSSION

### 3.1 Rapid qualitative assessment of HC‐DNA levels through acidification

An assay was developed to rapidly and qualitatively monitor HC‐DNA breakthrough across Emphaze AEX Hybrid Purifier during primary mAb clarification and may be used as a proxy for chromatin. pH adjustments, especially acidification, during mAb production are known to impact solution stability and potentially result in precipitation. The acidification of HCCF has been explored as a purification technique, in which a decreased pH precipitates HC‐DNA and other high‐molecular‐weight biological contaminants, thereby facilitating their removal.^22^ The assay considered during this study provides a means to obtain an estimate of HC‐DNA levels in real time during clarification without the delay often associated with more rigorous quantitative protocols by leveraging precipitation upon acidification and the resulting increase in turbidity.

**Figure 2:**
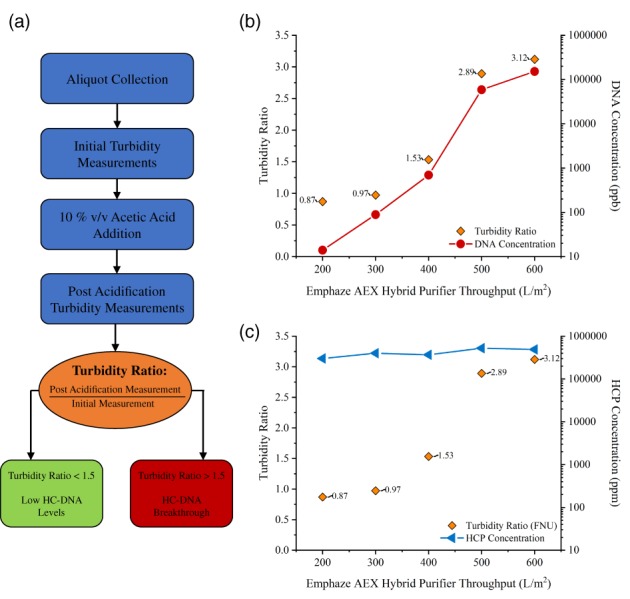
Rapid HC‐DNA screening using turbidity measurements. (a) Overview of turbidity measurements using acidification to facilitate the estimation of relative HC‐DNA removal. (b) Quantified HC‐DNA levels compared to formazin turbidimetric ratios. A ratio of∼1.5 using formazin turbidity measurements indicated an increase in HC‐DNA concentration by 2 orders of magnitude. (c) HCP concentrations in fractions collected at different Emphaze AEX Hybrid throughputs compared to formazin turbidity measurements. Lines depict trends only, not a fit to the data

As presented in Figure 2, the acidification assay implemented during this study involved the addition of approximately 20% by volume of 1 M acetic acid to the aliquots collected. The addition of acetic acid lowered the sample pH and induced precipitation, forming larger particles, and aggregates that were easily detected with conventional turbidity and DLS instruments. Clarification with Emphaze AEX Hybrid Purifier provided a reduction in HC‐DNA levels (Figure 2B) from 1.2 × 10^6^ ppb in the depth filtered pool to less than 100 ppb in the first 300 L/m^2^ throughput. The HCP concentration, however, was on the same order of magnitude in each fraction collected for all Emphaze AEX Hybrid Purifier throughputs considered (Figure 2C) and was comparable to the level in the starting depth filter pool (5 × 10^5^ ppm). As all samples collected had comparable HCP concentrations, the presence of HC‐DNA is hypothesized to lead to the formation of precipitate upon acidification.

To assess the utility of the turbidimetric assay, HC‐DNA levels were quantified via qPCR and correlated to the turbidimetric values before and after acidification. A qualitative indication of the HC‐DNA breakthrough was estimated by considering the ratio of turbidity measurements before and after acidification:

$$\text{Turbidimetric ratio}=\frac{\text{post acidification turbidity}}{\text{initial sample turbidity}.}$$

When trace HC‐DNA levels were present, little to no precipitation occurred upon acidification, and the sample turbidity remained relatively unchanged: the turbidity of an aliquot containing ∼60 ppb HC‐DNA was 0.92 FNU before acidification and 0.76 FNU after, resulting in a turbidity ratio slightly less than 1. In contrast, when higher levels of HC‐DNA were present, a discernable rise in turbidity was observed. At a HC‐DNA concentration of 6.2 × 10^5^ ppb, for example, turbidity increased from 5.9 FNU to 18.4 FNU upon acidification, yielding a turbidity ratio of approximately 3.

As presented in Figure 2B, higher turbidity ratios were observed as the HC‐DNA concentration increased, which provided a means to qualitatively assess HC‐DNA levels during clarification. A turbidity ratio near or less than 1.0 corresponded to a low HC‐DNA concentration measuring less than 100 ppb. As the FNU turbidity ratio reached and exceeded 1.5, the HC‐DNA concentration approached the >1 × 10^6^ ppb concentration present in unclarified material.

In practice, there are several methods available to assess turbidity. Formazin and nephelometric measurements are commonly used in biopharmaceutical applications during upstream processing to monitor clarification. Both nephelometric and formazin turbidity measurements make use of light scattering to assess and quantify clarification; however, the wavelength used differs between the two. Nephelometric turbidity measurements are commonly conducted using a broadband light source, whereas formazin turbidity relies on infrared wavelengths.^23^

**Figure 3:**
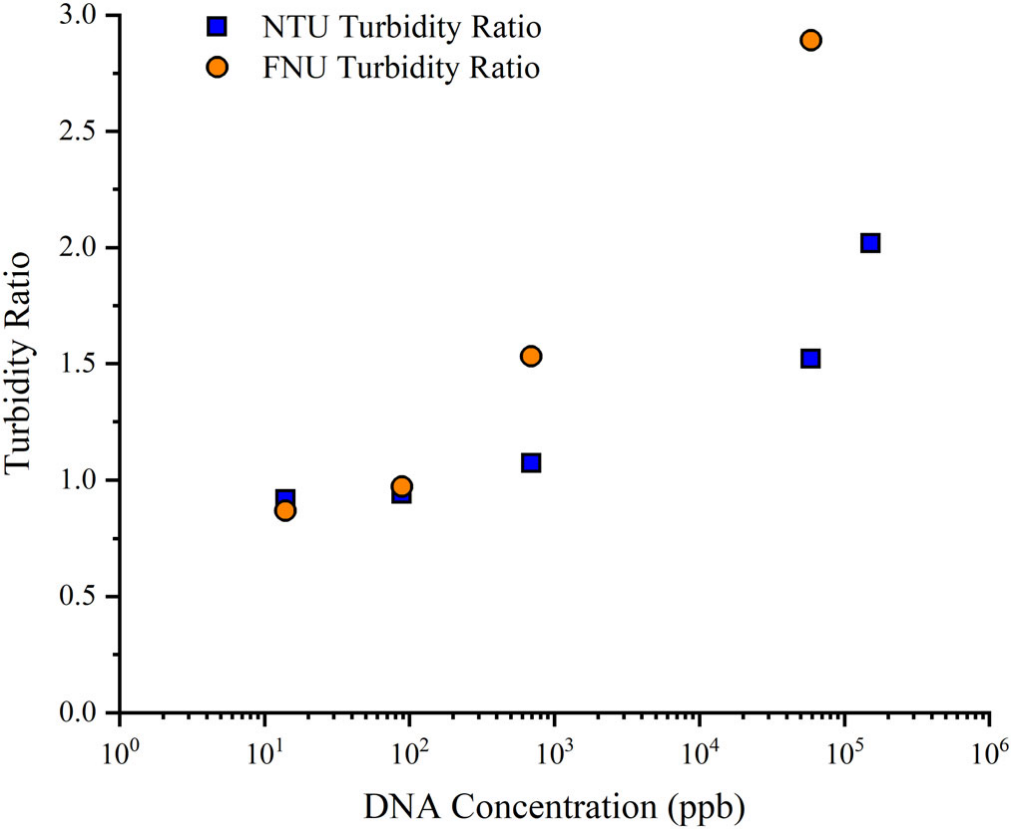
Comparison of turbidity measurements: formazin (FNU) vs. nephelometric (NTU) turbidimetric measurement techniques

Both turbidity measurement techniques provided a qualitative assessment of HC‐DNA levels, but with different levels of sensitivity. As evidenced in Figure 3, the use of formazin turbidity measurements in the qualitative assay provided higher sensitivity to HC‐DNA levels relative to nephelometric turbidity and enabled the distinction between 100s and 1000s of ppb of DNA. In contrast, ratios using nephelometric turbidity measurements did not provide a significant distinction between HC‐DNA concentrations of 100 and 1,000 ppb, where the turbidity ratio was approximately unity. With the acidification assay, turbidity ratios above 1 using nephelometric measurements indicated the onset of high levels of HC‐DNA as concentrations increased rapidly above this point.

The light source difference between these two measurements accounts for the difference in sensitivity. With HCCF typically exhibiting a pink to amber color, the use of an infrared light source in formazin measurements generally minimizes the impact of sample color on the turbidity results. In contrast, the white light of nephelometric measurements can be influenced by sample color.^23^, ^24^ Despite lower sensitivity, turbidity ratios obtained using nephelometric measurements provide a rapid, qualitative approach to screening and identifying the presence of appreciable levels of HC‐DNA.

**Figure 4:**
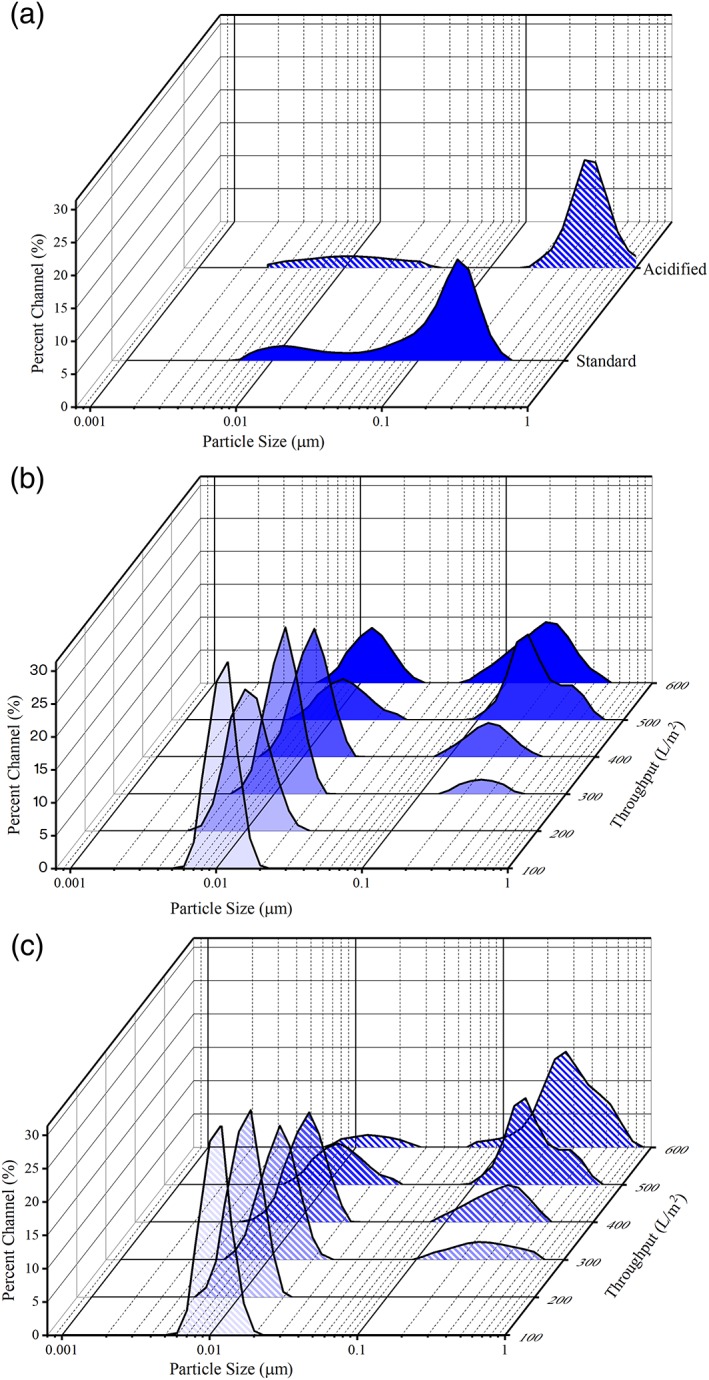
Dynamic light scattering intensity spectra. (A) Particle size distribution before and after acidification for material present in a pool clarified by depth filtration. (B) Particle size distribution of depth filtered material following clarification Emphaze AEX Hybrid Purifier. (C) Following acidification of aliquots, particle size distribution of depth filtered material further clarified with Emphaze AEX Hybrid Purifier

DLS can also provide information on the quality of CCCF. Owing to a diverse mixture of components such as host cells, HCPs, HC‐DNA, and the target protein, HCCF typically presents a wide and polydisperse particle size distribution. As shown in Figure 4A, the depth filtered feed evaluated during this study revealed particles ranging in size from ∼0.1 to 1 μm. Upon acidification, the DLS spectra shifted to contain a population of larger particles consistent with aggregation and precipitation. Following the clarification of HCCF via depth filtration and Emphaze AEX Hybrid Purifier, the particle size distribution became more defined, resulting in monodisperse populations at low throughputs. As illustrated in Figure 4, samples containing trace levels of HC‐DNA exhibited a monodisperse particle size distribution centered at approximately 0.01 μm before and after acidification, consistent with the size of protein‐based species, including mAbs.

The particle size distributions presented here were analyzed using an intensity weighted distribution. Intensity‐based results are highly sensitive to very small numbers of larger particles because the scattering intensity is proportional to the sixth power of the particle radius. The use of scattering intensity provides high sensitivity to the presence of larger materials such as HC‐DNA. When higher concentrations of HC‐DNA were present, a secondary particle size population appeared between 0.1 and 1 μm in the DLS spectra, characteristic of chromatin‐based species. Upon acidification, the magnitude of the larger sized particle population in the distribution increased, whereas that of the smaller particles declined. This result provides insight into the onset of contaminant breakthrough. Smaller particles do not scatter light to the same extent as larger particles and, therefore, can become obscured in the spectrum. This effect results in the apparent shift in the particle size distribution when larger material breaks through the media or when precipitation is induced. The DLS measurements and turbidity ratios acquired during the acidification assay were in agreement with one another. At low HC‐DNA concentrations (∼100 ppb), formazin turbidity ratios were less than one. Consistent with the turbidity readings, a monodisperse particle size distribution centered at approximately 0.01 μm was observed, which supported the hypothesis that protein is the dominant species in the filtrate. Elevated HC‐DNA levels were present at Emphaze AEX Hybrid Purifier throughputs above 300 L/m^2^, which resulted in a turbidity ratio greater than 1 and marked the appearance of an additional peak in the DLS spectra. For instance, at an Emphaze AEX Hybrid Purifier throughput of 200 L/m^2^, the turbidity ratio was 0.87, and a monodisperse particle size distribution was observed. When the throughput increased to 300 L/m^2^, the turbidity ratio increased to ∼1.0, and a second particle size population consistent with HC‐DNA began to develop in the DLS spectra.

### 3.2 Emphaze AEX hybrid purifier provides HC‐DNA reduction during primary clarification

**Figure 5:**
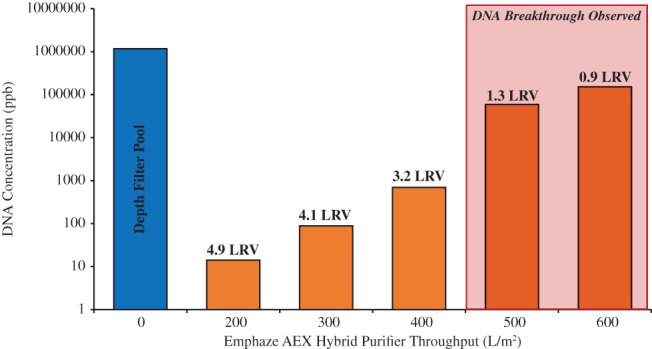
DNA reduction with depth filtration and at different Emphaze AEX Hybrid Purifier throughputs. Log reduction values (LRV) above Emphaze AEX Hybrid Purifier throughputs denote DNA reduction relative to the initial depth filter pool. The highlighted Emphaze AEX Hybrid Purifier samples at throughputs of 500 and 600 L/m^2^ had significantly higher DNA concentrations than the earlier fractions and were consistent with the findings of the turbidimetric assay: the turbidity ratios were found to be greater than 1.5

Traditional upstream clarification focuses primarily on the removal of insoluble, large debris, leaving soluble contaminants for downstream purification. For example, the use of a conventional depth filter train in this study reduced the turbidity of the CHO harvest to 26 NTU. Despite the turbidity reduction, depth filtration did not significantly remove soluble contaminants such as HC‐DNA, leaving ∼5 × 10^6^ ppb DNA in the filtrate pool. Inclusion of Emphaze AEX Hybrid Purifier following depth filtration provided a significant reduction in HC‐DNA‐related impurities without the need to adjust or add extraneous materials to the HCCF. As illustrated in Figure 5, relative to harvest clarified by depth filtration alone, the high charge capacity of Emphaze AEX Hybrid Purifier provided an approximately 5‐log reduction in DNA concentration to a throughput of 200 L/m^2^.

At higher throughputs, Emphaze AEX Hybrid Purifier continued to provide HC‐DNA removal; an approximately 3 log reduction in HC‐DNA was achieved at a throughput of 400 L/m^2^. Aliquots sampled at throughputs of 500 and 600 L/m^2^ were found to provide a 1 LRV of HC‐DNA, demonstrating that the capacity of the media was likely at or near exhaustion.

During clarification with Emphaze AEX Hybrid Purifier, the acidification assay was implemented to provide a rapid estimate of HC‐DNA levels. From Figure 2, the formazin turbidity ratios for Emphaze AEX Hybrid Purifier pools at throughputs of 200 and 300 L/m^2^ were 0.87 and 0.97, respectively, indicating low HC‐DNA levels. Quantification of HC‐DNA levels via qPCR showed that these ratios were consistent with DNA concentrations below 500 ppb. At a throughput of 400 L/m^2^, the turbidity ratio increased to ∼1.5, and an increase in HC‐DNA was observed. However, the DNA concentration was still relatively low at ∼3,000 ppb, and a reduction of ∼3 orders of magnitude in DNA was observed.

At a throughput of 500 L/m^2^, the turbidity ratio approximately doubled to 3, which corresponded to an HC‐DNA concentration of ∼2.4 × 10^5^ ppb. Only an approximately 1 log reduction in HC‐DNA concentration was observed at Emphaze AEX Hybrid Purifier throughputs greater than 400 L/m^2^, indicating that the purification media was at or near capacity.

Through the removal and reduction of HC‐DNA, Emphaze AEX Hybrid Purifier also provided a more consistent and standardized filtrate pool, as clearly demonstrated by the particle size distributions presented in Figure 4. Without the removal of HC‐DNA during primary clarification, CCCF typically presents a polydisperse particle size distribution, as shown in Figure 3A. When DNA began to break through Emphaze AEX Hybrid Purifier at throughputs above 200 L/m^2^, a secondary particle size population began to appear. Consistent with the quantitative measurements, at throughputs of 400 L/m^2^ or less, there was no apparent shift in particle size distribution upon acidification, and the smaller particle size population remaining dominant in the DLS spectra indicated that appreciable HC‐DNA breakthrough did not occur. At higher throughputs, the larger particle size population associated with HC‐DNA continued to evolve, approaching a distribution similar to that of the starting depth filter pool.

### 3.3 Lower HC‐DNA levels facilitate improved Protein A performance

**Figure 6:**
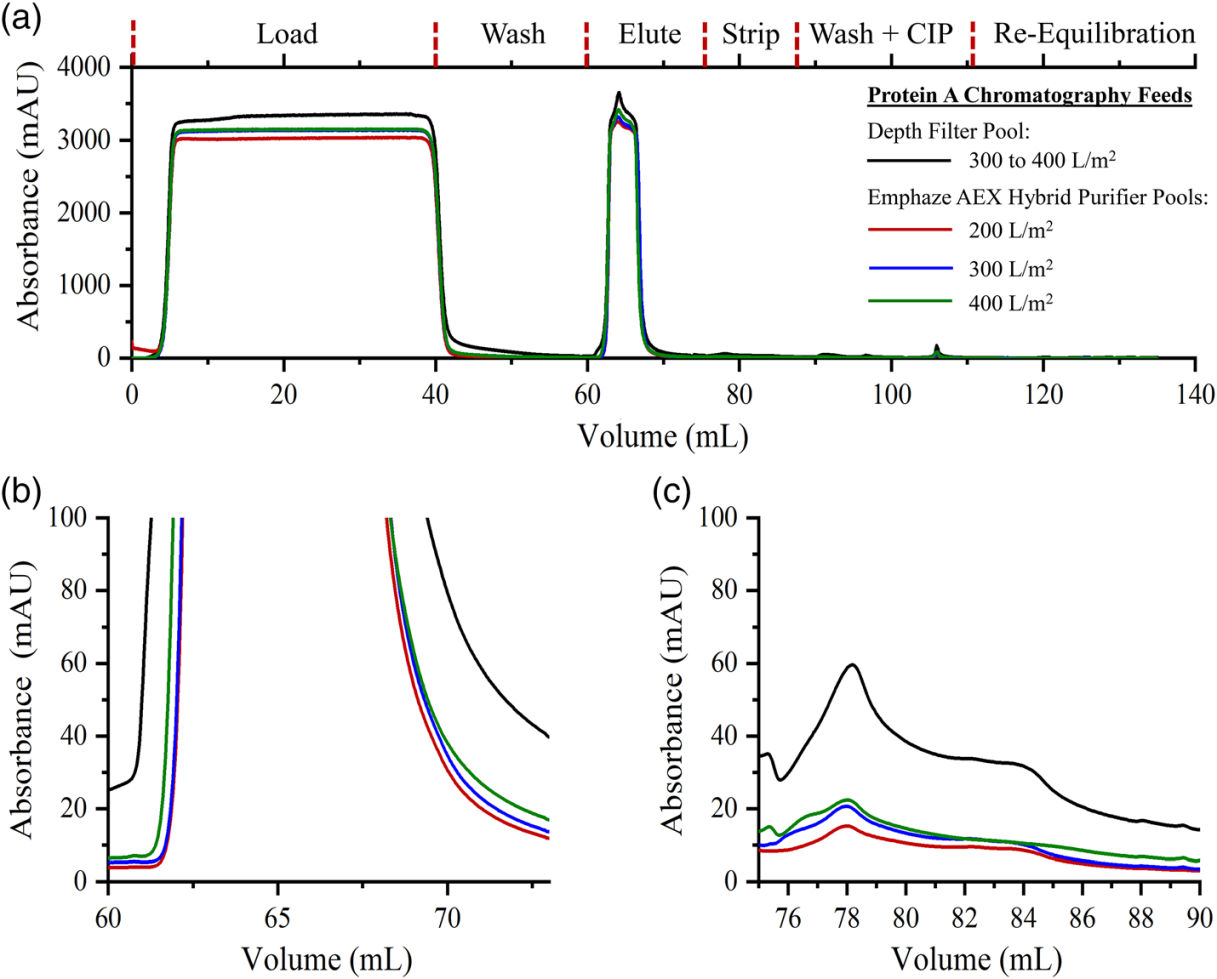
UV absorbance spectra at 280 nm acquired during Protein A chromatography: (A) UV absorbance over the course of the entire Protein A chromatography step, (B) profile during elution, and (C) UV absorbance of material during the column acid stripping

An improvement in Protein A performance was noted in the quality of the absorbance profiles during elution and the acid strip (Figure 6). When high levels of HC‐DNA were present, a broader absorbance peak was observed during the elution of tociluzimab, as evidenced in the elution profile of material from the depth filter pool. Material clarified by depth filtration alone had the highest HC‐DNA concentration and broadest Protein A absorbance peak, which spanned an elution volume of ∼10 mL. Protein A chromatography efficiency was improved when HC‐DNA levels were reduced and resulted in narrower tociluzimab elution profiles. For example, elution occurred over a volume of ∼8 mL when the HC‐DNA concentration was lowered to between 10 and 1,000 ppb. These narrower elution profiles provide a more concentrated eluate. As the capacity of the Emphaze AEX Hybrid Purifier was exhausted and the HC‐DNA levels increased, the elution UV trace profile became broader. For instance, when the HC‐DNA level was reduced to ∼10,000 ppb at an Emphaze AEX Hybrid Purifier throughput of 600 L/m^2^, an elution volume of ∼8.5 mL was required.

At lower HC‐DNA concentrations, a smaller UV absorbance signal was observed during the Protein A acid stripping. For material clarified by depth filtration alone, a peak absorbance of 60 mAU was observed during the acid stripping. In contrast, when HC‐DNA levels were lowered with Emphaze AEX Hybrid Purifier before Protein A chromatography, UV absorbance of between 10 and 20 mAU was observed during the acid stripping. Because of the reduction in HC‐DNA, less nonspecific binding to the Protein A ligands occurred, which resulted in reduced elution of impurities during the column acid stripping.

**Figure 7:**
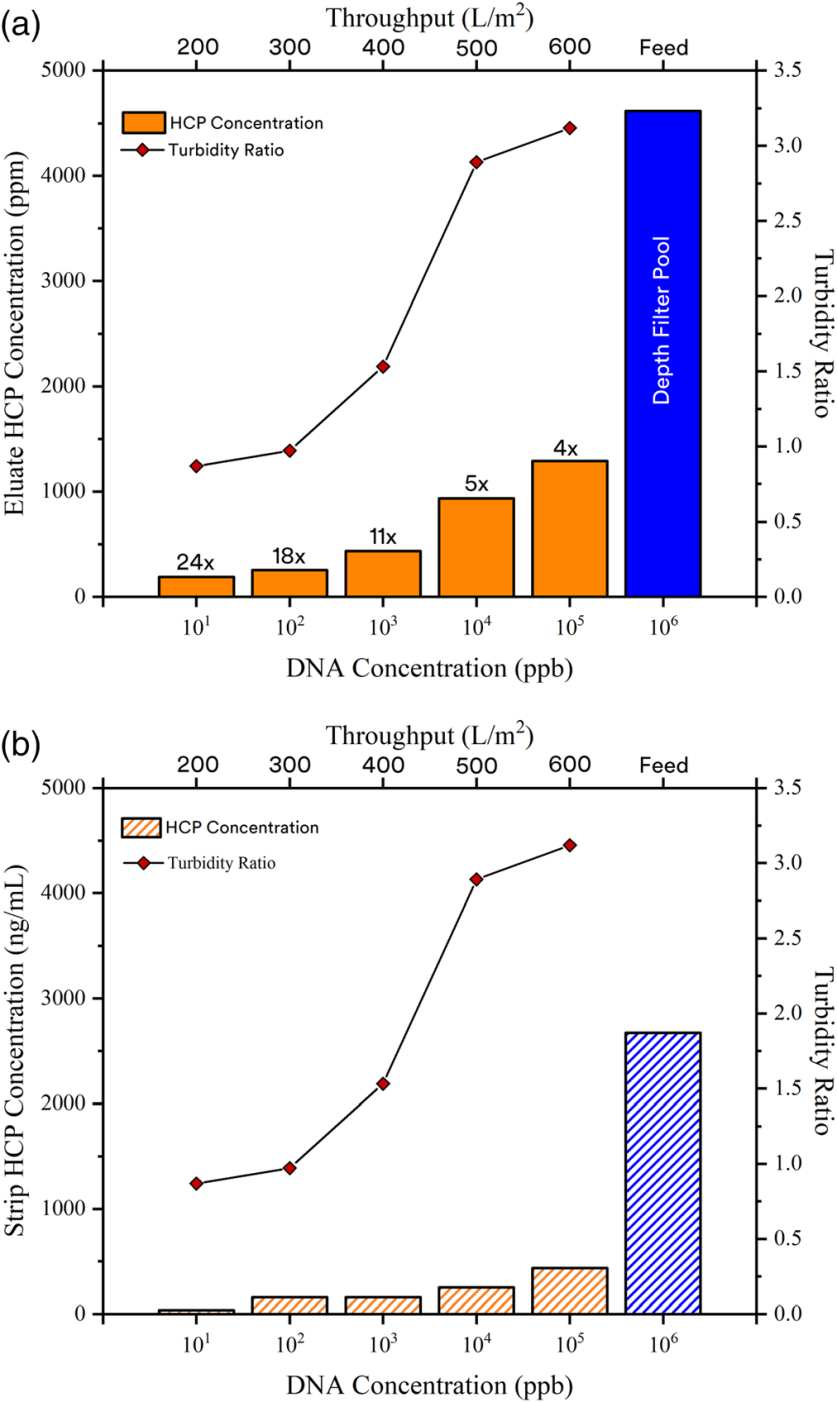
Protein A performance: reduction in HC‐DNA during primary clarification lowers nonspecific binding to and hitchhiking across the column. (A) HCP levels in the Protein A eluate pool when different levels of HC‐DNA were present in the affinity chromatography feed. (B) Concentration of HCP nonspecifically bound to the Protein A column as a function of HC‐DNA level in the feed material. Turbidity ratios derived from formazin measurements

In addition to the results observed in the UV absorbance profiles, the removal of HC‐DNA during primary clarification with Emphaze AEX Hybrid Purifier quantitatively improved HCP clearance by Protein A chromatography. As shown in Figure 7, the upstream reduction in HC‐DNA resulted in as much as a 24‐fold improvement in Protein A clearance of HCPs relative to conventional depth filtration.

At an Emphaze AEX Hybrid Purifier throughput of 200 L/m^2^, the DNA concentration in the Protein A load was reduced by five orders of magnitude: from 5 × 10^6^ ppb following depth filtration to ∼10 ppb. With an HC‐DNA reduction of this magnitude, a 24× improvement in HCP clearance across Protein A was obtained. Upon extending the Emphaze AEX Hybrid Purifier throughput to 400 L/m^2^, HC‐DNA was lowered to 1,000 ppb. At this HC‐DNA concentration, an ∼10× improvement in HCP reduction was still achieved with Protein A, which translated to ∼10× fewer impurities for downstream polishing steps to address. The improvement in HCP clearance in Protein A decreased as the HC‐DNA level in the Protein A load increased at higher AEX Hybrid Purifier throughputs.

A reduction in HC‐DNA levels during upstream clarification provided a benefit in terms of Protein A column regeneration.^25^ As presented in Figure 7, the elution of HCPs during the acid stripping of Protein A was quantified as a function of DNA concentration in the feed. Without a reduction in the HC‐DNA levels in the feed, a marked increase in the amount of nonspecifically bound HCPs was observed during Protein A regeneration. Following purification of a feed clarified by conventional depth filtration, acid washing of Protein A resulted in the elution of 500 to 1,000‐fold more HCPs relative to clarification where HC‐DNA was significantly reduced. Less HCP build up on the Protein A resin was observed when a 5‐log reduction in HC‐DNA concentration was achieved using Emphaze AEX Hybrid Purifier. After clarification with Emphaze AEX Hybrid Purifier, low HCP concentrations ranging from ∼40 to 400 ng/mL eluted during the acid stripping of the Protein A column.

**Figure 8:**
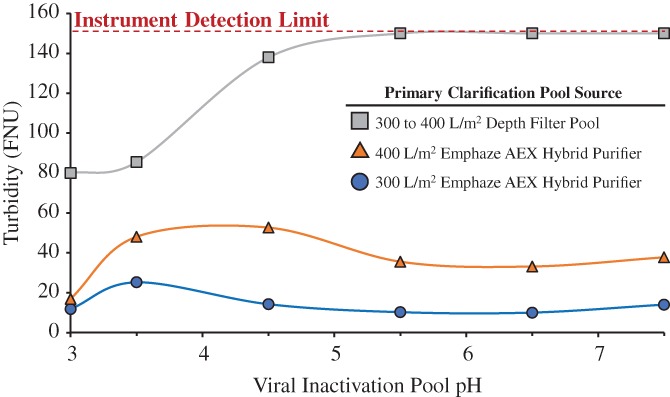
Viral inactivation pool turbidity levels during pH adjustment for harvest clarified by depth filtration alone and by Emphaze AEX Hybrid Purifier to throughputs of 300 and 400 L/m^2^. The dashed line at the top of the plot denotes the detection limit of the turbidity meter. Trendlines have been included for clarity and do not represent a fit to the data

Lower HC‐DNA levels were also found to reduce turbidity formation during pH adjustment of the Protein A eluate following viral inactivation. As illustrated in Figure 8, the turbidity of the material clarified by standard depth filtration exceeded the limit of detection during pH adjustment. The solubility of HC‐DNA and HCPs present in the Protein A eluate decreases during the pH adjustment, resulting in increased turbidity. Before further processing the turbidity must be reduced, typically through an additional filtration step, to protect and maintain the performance of downstream unit operations. Protein A chromatography performance was improved by using Emphaze AEX Hybrid Purifier during primary clarification, resulting in lower HC‐DNA and HCP levels in the eluate. The reduction in the Protein A eluate contaminant concentration resulted in decreased precipitation and consequently a smaller increase in turbidity upon pH neutralization of the viral inactivation pool.

## 4 CONCLUSION

When included in upstream purification, Emphaze AEX Hybrid Purifier provided a significant reduction in HC‐DNA levels compared to standard depth filtration. Emphaze AEX Hybrid Purifier reduced HC‐DNA concentration by approximately 5 logs. Therefore, Emphaze AEX Hybrid Purifier provides a cleaner, more standardized clarified fluid to enable improved HCP clearance during Protein A chromatography. For example, when a 5‐log HC‐DNA reduction was accomplished during primary clarification, Protein A was over 20× more effective at HCP removal. Furthermore, only an order of magnitude reduction in HC‐DNA using Emphaze AEX Hybrid Purifier was found to improve Protein A performance, providing ∼5× greater HCP clearance relative to that achieved by traditional primary clarification approaches.

Obtaining higher purity during upstream clarification also offered the potential for improved performance of downstream unit operations. This study demonstrated the detrimental impact of nonspecific binding of HC‐DNA and subsequent HCP hitchhiking on the performance of Protein A chromatography. As fewer impurities were nonspecifically bound to the Protein A resin when HC‐DNA was reduced during primary clarification, a shorter, less aggressive regeneration cycle may be possible. Reducing the time required for regeneration will allow improved cycle times and help minimize the exposure of the Protein A column to caustic conditions that are detrimental to the affinity ligands.^26^

A rapid qualitative acidification assay was developed as part of this study to facilitate the determination of HC‐DNA breakthrough across Emphaze AEX Hybrid Purifier and to provide a method to estimate HC‐DNA levels. A turbidimetric assay relying on measurements before and after acidification was developed to quickly screen for the presence of HC‐DNA and determine the capacity of Emphaze AEX Hybrid Purifier. Ratios of formazin turbidity measurements before and after acidification that were below 1.0 were found to indicate low HC‐DNA concentrations or HC‐DNA‐free samples. When the formazin turbidity ratios increased above 1, high levels of HC‐DNA were present, and these ratios were associated with breakthrough across Emphaze AEX Hybrid Purifier.

Supporting the results from the acidification assay, particle size measurements provided a means to identify significant quantities of DNA. In samples containing little or no HC‐DNA and chromatin‐related species, a monodisperse particle size distribution was observed centered at approximately 10 nm. The appearance of a secondary particle size population of approximately 100 nm indicated the presence of HC‐DNA‐ and chromatin/DNA‐related impurities. Furthermore, the addition of acetic acid was demonstrated to induce the precipitation of HC‐DNA and skew the distribution to larger particles. The use of this assay provides a tool for assessing HC‐DNA and chromatin‐related impurity levels, facilitating the identification of DNA breakthrough across Emphaze AEX Hybrid Purifier during purification.
